# Combined lethal intoxication involving 2-methyl-2-butanol

**DOI:** 10.1007/s00414-026-03900-3

**Published:** 2026-07-01

**Authors:** Martin Dokter, Paul Hagen, Julia Wudtke, Ricarda Kegler, Britta Bockholdt, Giovanni Talarico

**Affiliations:** 1 Institute of Legal Medicine, University Medical Centre of Greifswald, Kuhstraße 30, Greifswald, 17489 Germany; 2 Institute of Legal Medicine, University Medical Centre of Rostock, St.-Georg-Straße 108, Rostock, 18055 Germany

**Keywords:** Tert-amyl-alcohol, Intoxication, HS-GC-FID, Ethanol

## Abstract

The aim was to investigate and clarify the cause of death of a 26-year-old male found dead in his apartment. It was known that the individual used drugs of abuse. In the apartment, the police found amongst others a “brown powder” and a glass bottle labeled with “2-methyl-2-butanol” (2m2b). Autopsy and chemical analyses were performed. The autopsy revealed unspecific morphological signs of an intoxication (cerebral swelling and cyanosis, pulmonary edema, full urine bladder). Headspace gas chromatography with flame ionization detection (HS-GC-FID) was used to determine 2m2b and ethanol. Concentrations of 245 mg/L and 225 mg/L 2m2b could be detected in the femoral vein blood specimen and urine, respectively. Ethanol was detected below the level of quantification (0,2 g/L) in both matrices. The detected blood concentration of 2m2b was more than twice as high as the 2m2b concentration described as comatose in a previous published case report. However, in the presented case further analysis revealed toxic concentrations of methamphetamine and venlafaxine and also identified a previous intake of heroin and diazepam. Thus, a combined drug intoxication was assumed as cause of death. Little is known about the abuse of 2m2b as an alternative to ethanol. Since 2m2b is widely available, cheap, more potent than ethanol, while lacking its hangover effects and is likely not detected by routine HS-GC-FID methods for the detection of blood ethanol concentrations, it may be of forensic relevance.

## Introduction

Historically, 2-methyl-2-butanol (2m2b; tert-amyl-alcohol; amylene hydrate) was used as an anesthetic due to its sedative and hypnotic effects [1], but is without any medical importance today. However, it is still used in the chemical industry, e.g. as a solvent for different coatings or reagent for the synthesis of pharmaceuticals. More recently, a few reports about recreational use of 2m2b in Germany, Poland and the USA emerged [2–5]. While having a higher potency than ethanol [6], its similar effects to the central nervous system [7] make 2m2b a candidate as a recreational drug. This is further increased by questionable “benefits” as a lacking hangover, a decreased chance of detection by screening tests such as common breathalyzers [4] and an easy access [8]. Recreational use was also confirmed by web search of known drug scene web sites [9, 10]. Given its properties, a misuse of 2m2b as a knockout agent, especially concealed with ethanol consumption, is imaginable. However, literature research on 2m2b did not reveal any information about this.

Compared to ethanol, little is known about the toxicity and metabolism of 2m2b in humans. Preclinical data in rats and rabbits show a higher toxicity (LD_50_ = 1–2 g/kg BW) in comparison to ethanol (LD_50_ = 7–9 g/kg BW) [6]. The metabolic pathway in rats was shown to involve glucuronidation [11] and oxidation to 2-methyl-2,3-butanediol [12]. Currently, literature research revealed only one case of 2m2b intoxication with reported analytical data. In this case, a patient who ingested 2m2b recreationally was admitted to the hospital with deep coma and acute respiratory failure. In the blood sample collected at admission, a 2m2b concentration of 83 mg/L was detected [4].

Beside recreational use, 2m2b could also be ingested in very little amounts after inhalation of gasoline vapours containing t-amyl methyl ether. Demethylation of t-amyl methyl ether leads to the formation of formaldehyde and 2m2b [13].

## Case report

**Fig. 1:**
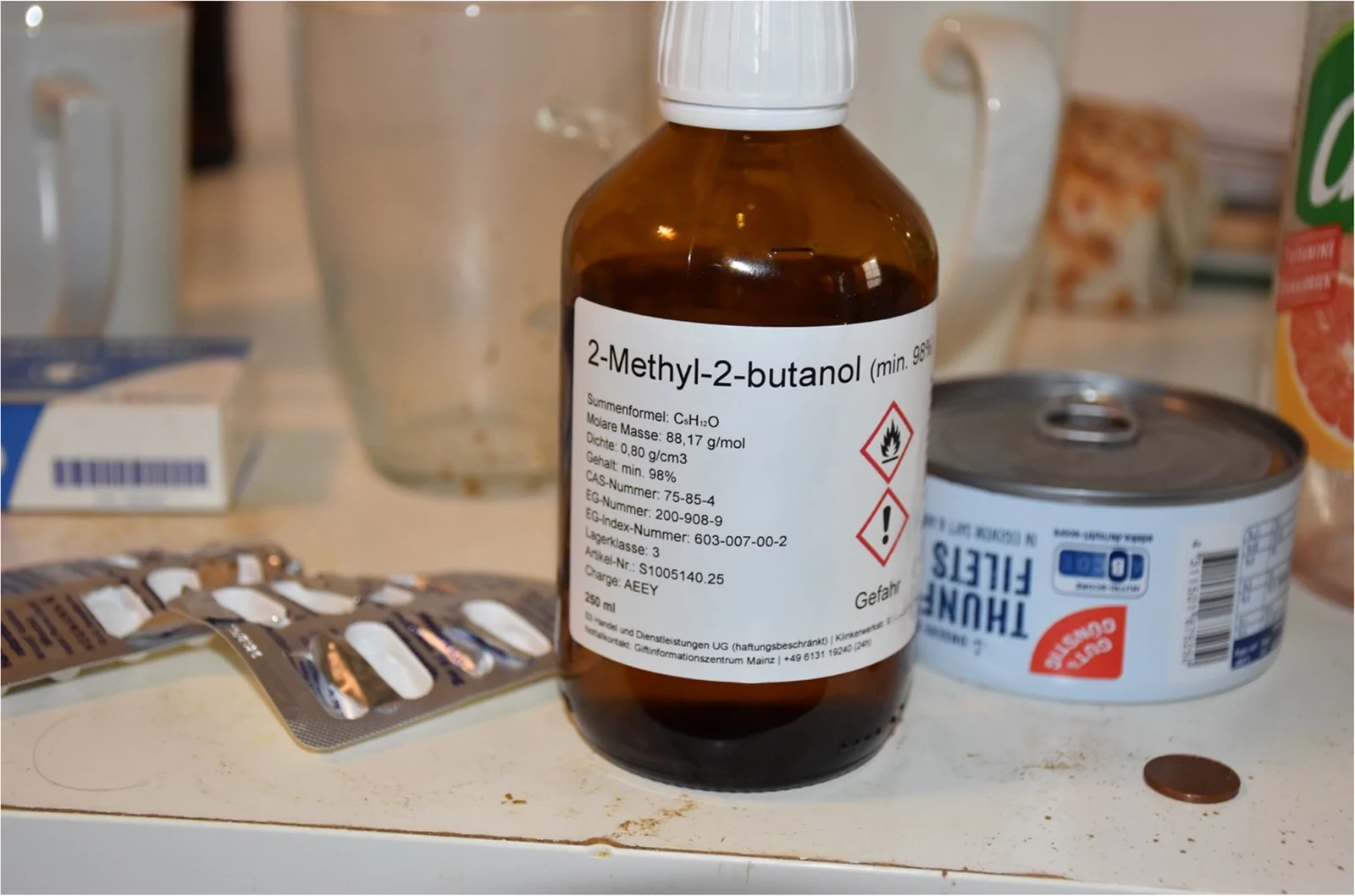
Bottle with 2m2b found by the police in the apartment of the deceased. (source: Polizeipräsidium Neubrandenburg, Kriminalpolizeiinspektion Anklam, Fachkommissariat 7 [KDD Stralsund])

A 26-year-old man known to be abusing drugs was found dead in his apartment. In the apartment, the police found psychotropic medication (venlafaxine 75 mg, pregabalin 300 mg, quetiapine 400 mg), a sniffing tube, a “brown powder” and a typical zipping bag with “little brown particles”, both detected as ecstasy by a police substance test. Furthermore, a glass bottle labeled with “2-methyl-2-butanol” (2m2b) was found (see Fig. 1). The autopsy revealed no injuries but unspecific signs of intoxication: pulmonary edema, full urinary bladder (800 mL), cerebral cyanosis. However, there was no diagnosable cause of death morphologically. Chemical analyses concerning drugs and ethanol were performed using routine GC-MS, HPLC-DAD and HS-GC-FID methods (see results). In addition, a HS-GC-FID method for the detection of 2m2b was developed.

## Experimental analysis of 2m2b

### Reagents and materials

2m2b (purity 99.13%) for calibration was purchased from Dr. Ehrenstorfer (LGC, Augsburg, Germany) and 2m2b (purity 99%) for quality controls was bought from Sigma (Merck, Darmstadt, Germany). Tert-butanol as internal standard (IS) was bought from Merck (Merck, Darmstadt, Germany).

Blank blood control samples (free of ethanol and other alcohols) were purchased from Medichem (MEDICHEM Diagnostic, Steinenbronn, Germany).

### Preparation of standards, calibrators and quality controls

Calibrators (10, 20, 50, 100, 200, 500 mg/L) were prepared by corresponding dilution with water. Quality controls (QC) with two different 2m2b levels (100 und 500 mg/L) were prepared by spiking blank blood controls with adequate amounts of 2m2b. Blank blood controls were also used as negative control.

### Sample preparation

Body fluids and organ materials were collected during autopsy (femoral vein blood, heart blood, urine, kidney, liver, bile, muscle, adipose tissue as well as contents of stomach, duodenum and colon) and stored at -20 °C.

100 µL of liquid samples (incl. QCs and calibrators) or 100 mg of organ materials and 1000 µL of aqueous IS (80 mg/L tert-butanol) in 20 mL headspace vials were analyzed by headspace gas chromatography with flame ionization detector (HS-GC-FID).

### Methods

Quantitation of 2m2b was carried out at the Institute of Legal Medicine Greifswald by an in-house method using a HS-GC-FID system from Perkin Elmer (Shelton, USA) consisting of a TurboMatrix HS-110 automatic headspace sampler, a Clarus 690 gas chromatograph with a flame ionization detector and an Elite-BAC2 Advantage (30 m x 0.32 mm ID x 0.60 μm film thickness) capillary column. The headspace method parameters were as follows: carrier gas pressure (nitrogen) 130 kPa, oven temperature 75 °C, thermostatting time 16.5 min, needle temperature 100 °C, transfer line temperature 110 °C, pressurization time 1.5 min, injection time 0.03 min. The following GC oven program was used: 50 °C (2 min) > 45 °C/min > 110 °C (4 min).

For further confirmation of 2m2b via retention time comparison, two similar HS-GC-FID systems with different columns were used: an Elite-BAC1 Advantage (30 m x 0.32 mm I.D. x 0.60 μm) capillary column and a packed column with 0.2% carbowax 1500 on 60/80 CarboBlack C (6 ft x 1/8 in x 2.1 mm).

### Validation

Selectivity, specificity and linearity of calibration were validated in accordance with the Guidelines of the Society of Toxicological and Forensic Chemistry [14] using the Valistat 2.0 software (Arvecon, Walldorf, Germany). Accuracy was determined with self-made QCs. Due to the lack of appropriate proficiency tests, accuracy could not be further verified.

## Results

### 2m2b analysis

**Fig. 2:**
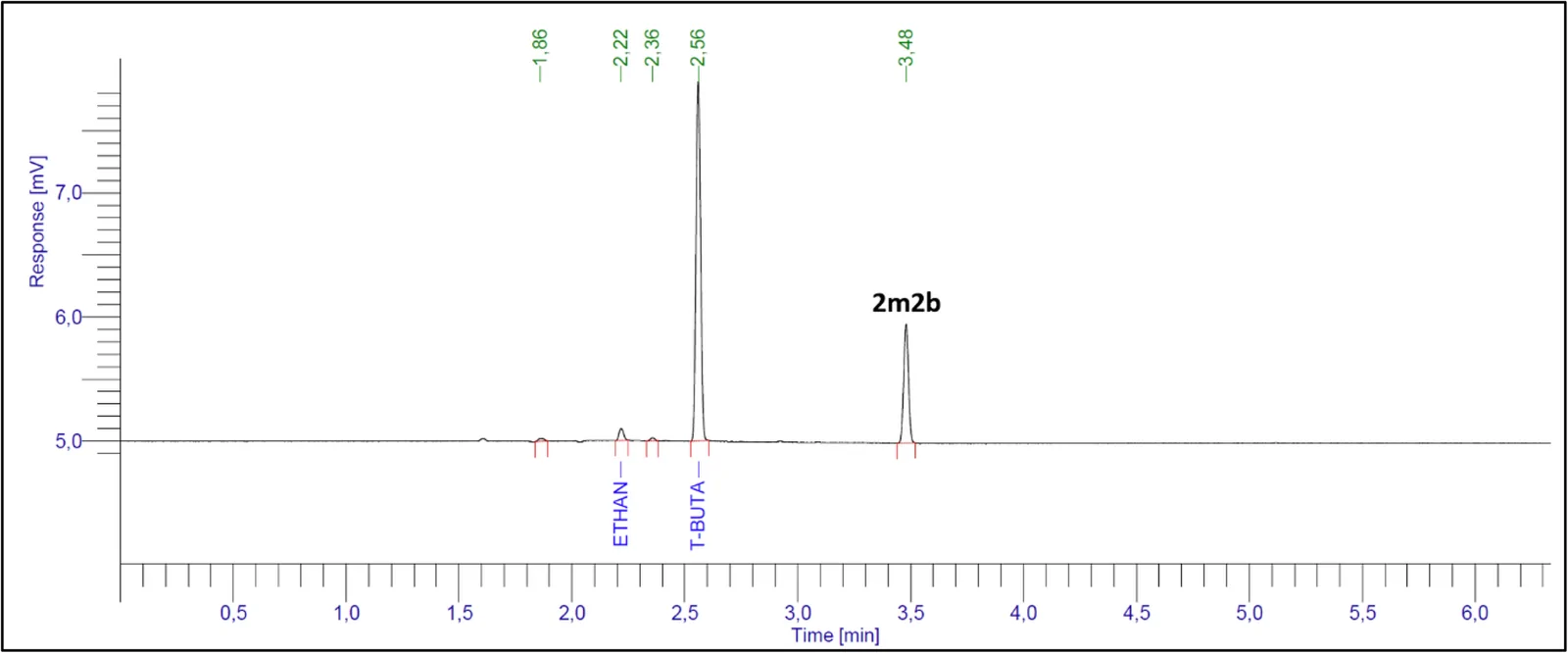
Chromatogram of HS-GC-FID analysis of 2m2b in femoral vein blood (245 mg/L)

Identification of 2m2b via retention times was confirmed on three HS-GC-FID systems with three different columns. 2m2b concentrations detected in postmortem samples ranged from 111 mg/L – 1006 mg/L. In femoral vein blood, heart blood and urine 2m2b concentrations were detected 245 mg/L (Fig. 2), 215 mg/L and 225 mg/L, respectively. In the gastric content (570 mL), a concentration of 1006 mg/L was found, indicating about 573 mg of 2m2b left in the stomach at the time of the autopsy. A comprehensive overview of detected 2m2b in all matrices can be found in Table 1.

**Table 1:** 2m2b concentrations in various postmortem matrices

Matrix	Concentration
Femoral blood	245 mg/L
Heart blood	215 mg/L
Urine	225 mg/L
Kidney	135 mg/L
Liver	213 mg/L
Bile	279 mg/L
Muscle	116 mg/L
Adipose tissue	118 mg/L
Gastric content	1006 mg/L
Duodenal content	323 mg/L
Colon content	111 mg/L

Besides minor peaks for acetaldehyde and aceton, no unusual peaks were detected that could indicate the presence of 2m2b in the postmortem samples. However, a carryover of 2mb2 into the second sample was noticed during the routine ethanol measurement using a packed column with isothermal oven program (manifesting as significant peak tailing of the internal standard). On the other hand, chromatograms obtained during ethanol measurements with a capillary column using a temperature gradient did not show any carryover.

### Other analyses

Further toxicological analyses (performed in the Institute of Legal Medicine Rostock) in femoral vein blood, urine and gastric content revealed methamphetamine (femoral blood: approx. 1700 ng/mL, urine: approx. 4500 ng/mL), amphetamine (femoral blood: 100 ng/mL, urine: approx. 1200 ng/mL) and venlafaxine (femoral blood: 1900 ng/mL). Moreover, morphine (femoral blood: 27 ng/mL, urine: 710 ng/mL), codeine (urine: 72 ng/mL) and 6-monoacetylmorphine (urine: 220 ng/mL) as well as diazepam (femoral blood: 84 ng/mL) and nordazepam (femoral blood: 23 ng/mL) were detected (see Table 2). Ethanol concentrations were below the level of quantification (femoral blood: approx. 0.03‰, urine: approx. 0.04‰).

**Table 2:** Results of the toxicological analyses. + = detected, but not quantified, n.a. = not analyzed, n.d. = not detected

Analyte	Method	Blood (ng/mL)	Urine (ng/mL)	Gastric cont.
Methamphetamine	GC-MS	approx. 1700	approx. 4500	+
Amphetamine	GC-MS	100	approx. 1200	+
Morphine	GC-MS	27	710	n.d.
Codeine	GC-MS	n.d.	72	n.d.
6-MAM	GC-MS	n.d.	220	n.d.
Venlafaxine	HPLC-DAD (urine: GC-MS)	1900	+	+
Diazepam	HPLC-DAD	84	n.a.	n.d.
Nordazepam	HPLC-DAD	23	n.a.	n.d.

## Discussion

In the presented case, the toxicological analyses revealed potentially toxic concentrations of methamphetamine and venlafaxine as well as the intake of heroin and diazepam. Analyses also revealed expected metabolites of these substances, i.e. amphetamine as metabolite of methamphetamine, 6-MAM as metabolite of heroin, which was not detected itself, nordazepam as a metabolite of diazepam. Additionally, codeine was detected in urine. This could either indicate an intake of a codeine preparation in the past or more likely show the intake of illicit heroin which usually contains codeine deriving from opium used for illegal heroin production. Furthermore, the ingestion of 2m2b was shown. Considering the only published clinical case of a severe 2m2b intoxication with analytical data [4], the presented 2m2b concentration is assessed as lethal. Thus, this case describes the first lethal intoxication involving 2m2b with corresponding analytical data. The cause of death is therefore a combined drug intoxication of 2m2b, methamphetamine and venlafaxine. While the temporal course of the intoxication is unknown, the full urinary bladder suggests a prolonged phase of agony and for this reason the intake of 2m2b was a certain time before death.

Little is known about the pharmacologic properties of 2m2b. Presented data and the solubility in water (logP 2m2b: 0.89 / logP ethanol: -0.31) [15] show that 2m2b is more lipophilic and underlies a higher distribution in adipose tissue than ethanol. This also suggests a higher accessibility of 2m2b to the central nervous system. It is known that the relation of narcotic and hypnotic influence of alcohols is associated with their lipophilicity, i.e. hypnotic effects dominate in more lipophilic alcohols [16]. This likely leads to the higher potency of 2m2b compared to ethanol with regard to their hypnotic effects. However, potency factors should be determined and applied cautiously. The suggested 20 times higher potency of 2m2b as cited by previous authors [5, 6], derives from a publication of Brandenberger et al. [16]. Brandenberger et al. compare doses of 2–4 g 2m2b and 100 g ethanol which either induce sleep (2m2b) or “put an adult to sleep” (ethanol). However, Brandenberger et al. do not further elaborate on this comparison, i.e. to what extent sleep induction is performed by both substances. The effective dose of 2m2b is confirmed in a historical study in which typical doses of up to 4 g 2m2b were observed to be sleep inducing and following which only one of 40 patients reported inebriation effects [17]. Further, the author states that sleep came quickly, but patients could be easily awakened being clear-headed at any time. In contrast, comparable isolated hypnotic effects are not to be expected after an intake of 100 g ethanol which would be associated with a significant degree of inebriation. With regards to the inebriation effects, past dose comparisons therefore seem to allow only a vague estimate of a potency factor and the above-mentioned relation of narcotic and hypnotic effects in different alcohols should be considered. More recent publications by Rusiecka et al. and Daub et al. [5, 6] apply the potency factor of 20 to volumes of 2m2b and vodka (typically 40 Vol-% ethanol). Thus, Rusiecka et al. equate 7,5 mL 2m2b (about 6 g) with about 150 mL vodka (about 48 g ethanol) regarding inebriation effects of both substances, i.e. warm alcohol glow, a feeling of well-being, increased talkativeness. Based on the calculated ethanol content of vodka the example would lead to an 8 times higher potency of 2m2b which is equivalent to the factor of the LD50 ratio of 2m2b and ethanol in rats and rabbits [6]. Considering this, previous estimates of an overall 20 times higher potency appear rather overestimated and we suggest to assume a lower potency factor with regards to inebriation effects and ensuing recreational use. However, with the available data a dose-effect relationship cannot be established and due to the different pharmacologic properties, comparisons with ethanol appear unfeasible, especially in regard to potentially toxic or lethal doses and resulting blood levels of 2m2b.

The published literature from Europe and the USA suggests an increasing importance of 2m2b as a recreational drug [2–5]. 2m2b is not routinely detected with the HS-GC-FID method during ethanol analysis for detecting the blood ethanol concentration in DUI-cases in our laboratory. Therefore, the prevalence of 2m2b abuse in our region is unclear. However, since 2m2b shows a carryover on one of our HS-GC-FID systems used for routine ethanol measurements, a suspicious chromatogram would have led us to further analyses.

## Conclusion

To assess the importance of 2m2b abuse in the general population routine measurement of ethanol in DUI-cases should be adapted to include 2m2b. Further, pharmacological studies should be employed for better interpretation of intoxication cases.
